# High expression of TROP2 correlates with poor prognosis in pancreatic cancer

**DOI:** 10.1038/sj.bjc.6604677

**Published:** 2008-09-23

**Authors:** D Fong, P Moser, C Krammel, J M Gostner, R Margreiter, M Mitterer, G Gastl, G Spizzo

**Affiliations:** 1 Department of Hematology and Oncology, Innsbruck Medical University, Innsbruck 6020, Austria; 2 Tyrolean Cancer Research Institute, Innsbruck 6020, Austria; 3 Department of Pathology, Innsbruck Medical University, Innsbruck 6020, Austria; 4 Department of General and Transplant Surgery, Innsbruck Medical University, Innsbruck 6020, Austria; 5 Department of Oncology and Hematology, Franz Tappeiner Hospital, Merano 39012, Italy

**Keywords:** TROP2, GA733, pancreatic cancer, targeted therapy

## Abstract

Pancreatic cancer is one of the most devastating human malignancies. Despite considerable research efforts, it remains resistant to almost all available treatment regimens. The human trophoblast cell-surface antigen, TROP2, was found to be strongly expressed in a variety of human epithelial cancers, correlating with aggressiveness and poor prognosis. TROP2 antigen expression was investigated retrospectively by immunohistochemistry in paraffin-embedded primary tumour tissue samples from a series (*n*=197) of consecutive patients with pancreatic adenocarcinoma. Survival was calculated using Kaplan–Meier curves. Parameters found to be of prognostic significance in univariate analysis were verified in a multivariate Cox regression model. TROP2 overexpression was observed in 109 (55%) of 197 pancreatic cancer patients and was significantly associated with decreased overall survival (*P*<0.01). By univariate analysis, TROP2 overexpression was found to correlate with the presence of lymph node metastasis (*P*=0.04) and tumour grade (*P*=0.01). Furthermore, in the subgroup of patients treated surgically with curative intent, TROP2 overexpression significantly correlated with poor progression-free survival (*P*<0.01). Multivariate analyses revealed TROP2 to be an independent prognosticator. These findings suggest for the first time that TROP2 could be a novel prognostic biomarker for pancreatic cancer. Targeting TROP2 might be a useful treatment approach for patients with pancreatic cancer overexpressing this cell-surface marker.

Pancreatic cancer is a fatal disorder. At the time of diagnosis, most patients already have locally advanced or metastatic disease excluding surgical resection (Yeo *et al*, 1997). Treatment regimens followed at present are ineffective and do not prolong patient survival significantly. Survival rates for the 15–20% of patients who have resectable disease at first presentation are disappointing, depending on the status of resection margins and surrounding lymph nodes. Moreover, the prognosis is poor even for those who undergo complete (R0) resection. The 5-year overall survival rate for all stages is approximately 5%, whereas after radical pancreaticoduodenectomy it has been reported to range from 25 to 30% (Sohn *et al*, 2000; Jemal *et al*, 2005). To date, there are no validated prognostic indicators for identifying patients with non-metastatic disease and good or poor prognosis. As a consequence, there is an urgent need for novel prognostic biomarkers as well as for therapeutic target structures in pancreatic cancer.

In the past, we found GA733-encoded proteins (EpCAM, TROP2) to be prognostic for a variety of epithelial cancers (Gastl *et al*, 2000; Varga *et al*, 2004; Fong *et al*, 2008). GA733 is a member of a family of at least two closely related genes (*GA733-1*; *TACSTD2* and *GA733-2*; *TACSTD1*) that share sequence homology with both thyroglobulin type I and interleukin-2 receptors. The integral glycoprotein EpCAM is encoded by the *GA733-2* gene functions as a homotypic intercellular adhesion molecule (Litvinov *et al*, 1994), and has been targeted by antibody-mediated immunotherapy (Riethmuller *et al*, 1998; Ruan *et al*, 2003).

The human trophoblast cell-surface antigen, TROP2 (also termed GA733-1), is a transmembrane glycoprotein found to be expressed at high levels by various types of human carcinomas (Fradet *et al*, 1984; Stein *et al*, 1993a; Nakashima *et al*, 2004). In contrast, normal epithelial tissues show little or no TROP2 expression (Stein *et al*, 1993a; Zhang *et al*, 1997). TROP2 is encoded by the *GA733-1* gene, which is intronless, formed by exon-shuffling and retroposition of the *GA733-2* gene through mRNA intermediate (Linnenbach *et al*, 1993). Recently, TROP2 antigen expression in colorectal and squamous cell carcinomas of the oral cavity was shown to correlate with tumour aggressiveness and poor prognosis (Ohmachi *et al*, 2006; Fong *et al*, 2008). Moreover, Wang *et al* (2008) were able to identify TROP2 as an oncogene and an attractive therapeutic target in colon cancer. These findings indicate a potential role for TROP2 in tumour development and progression. In this retrospective study, we evaluated TROP2 antigen expression and its correlation with clinicopathologic features in pancreatic cancer.

## Materials and methods

### Patients and tissue samples

This study was conducted according to the regulations of the local Ethics Committee and Austrian law. A total number of 197 consecutive patients with pancreatic ductal adenocarcinoma, from whom formalin-fixed, paraffin-embedded tissue samples were available, diagnosed between 1990 and 2006 at the Department of Pathology, Innsbruck Medical University, were included in this retrospective study. Patients with other pancreatic malignancies, such as intraductal papillary mucinous adenocarcinoma, acinar cell carcinoma and malignant endocrine tumours, were excluded. All tumour specimens were reclassified on hematoxylin-and-eosin-stained slides, and histological type and tumour grade were reassessed by a pathologist (PM) using standard diagnostic criteria. Clinical data were obtained by reviewing the charts and contacting the physicians in charge. Tumours were histologically classified according to the WHO classification and staged according to the tumour node metastasis classification (Sobin and Fleming, 1997). Overall survival was defined as the period of time from initial diagnosis to death or last contact, that is, date of last follow-up visit. Progression-free survival was defined as the time from surgery with curative intent to appearance of disease recurrence or evidence of new lesions detected by computed tomography.

### Immunohistochemistry

**Figure 1:**
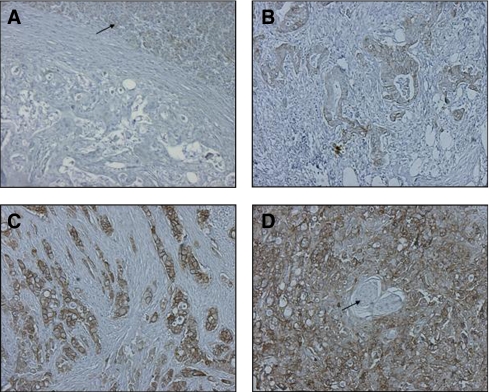
TROP2 immunostaining in pancreatic tissue, original magnification × 100. (**A**) TROP2-negative tumour sample (score 0), normal pancreatic tissue (arrow) showing weak (score 2) immunostaining. Pancreatic ductal adenocarcinoma with moderate (score 6) (**B**) and strong (score 12) (**C** and **D**) TROP2 expression, note the TROP2-negative islets of Langerhans (arrow).

TROP2 expression was determined by immunohistochemistry using a purified goat polyclonal antibody detecting the recombinant human TROP2 extracellular domain at a dilution of 1 : 50 (AF650, R&D Systems, Inc., Minneapolis, MN, USA) as described earlier (Fong *et al*, 2008). Colon carcinoma samples with various TROP2 expression levels (no, low, moderate, or high expression) were used as positive and negative controls. In addition, 10 samples of normal pancreatic tissue were collected from patients undergoing pancreatic surgery at Innsbruck Medical University. TROP2 antigen overexpression was evaluated by two independent observers (PM and DF) using light microscopy in a blinded manner. Discordant cases were reevaluated on a double-headed microscope to achieve a consensus. Antigen expression was defined as the presence of specific staining on surface membranes of tumour cells. TROP2 overexpression was evaluated for each tissue sample by calculating a total immunostaining score as the product of a proportion and intensity score. The proportion score described the estimated fraction of positive-stained tumour cells (0, none; 1, <10%; 2, 10–50%; 3, 51–80%; 4, >80%). The intensity score represented the estimated staining intensity (0, no staining; 1, weak; 2, moderate; 3, strong). The total score ranged from 0 to 12. As described earlier, TROP2 ‘overexpression’ was defined as a total score of more than 4 (Fong *et al*, 2008). Representative micrographs of tumours with predominant membranous staining of TROP2 are shown in Figure 1.

### Statistical analysis

Statistical analysis was performed using the Statistical Package of Social Sciences (SPSS, version 10.0, Chicago, IL, USA). Correlations between TROP2 expression and clinicopathological variables were assessed with the χ^2^-test. Survival rates were calculated using the Kaplan–Meier method and compared by means of the log-rank test. Follow-up time was censored if the patient was lost during follow-up. Factors with prognostic significance in the univariate models were further evaluated in a multivariate Cox regression model. For all analyses, a *P*-value of less than 0.05 was considered statistically significant.

## Results

### Patient characteristics

Demographic data and tumour characteristics are summarised in Table 1. At the time of last clinical follow-up (February 2008), 155 (79%) patients out of the total group had died. Median overall survival was 9 months (range, 1–68). Most patients (68%) had undergone primary surgical intervention, whereas 54 (27%) patients were considered inoperable (9 cases unknown). Palliative surgery included palliative bypass or endoscopic bile duct stenting. If indicated, patients received standard gemcitabine-based chemotherapy according to their clinical stage and performance status. In 17 patients, exact staging according to UICC was not feasible.

### Immunohistochemistry

As reported earlier, islets of Langerhans displayed no TROP2 staining (Stein *et al*, 1993a), whereas in normal pancreatic epithelium TROP2 expression was weak to moderate (Figure 1). In contrast, moderate-to-strong homogeneous membranous expression of TROP2 was detected in 109 (55%) of 197 carcinoma specimens. Strong TROP2 expression (score 9–12) was found in 57 (29%) of 197 cases, moderate expression (score 6–8) in 52 (26%) cases, weak expression (score 1–4) in 76 (39%) cases and absent expression (score 0) in 12 (6%) of 197 cases. Thus, according to our earlier defined criteria (Fong *et al*, 2008), TROP2 was found to be overexpressed in 109 (55%) of 197 cases.

### Clinicopathological variables and patient survival

**Figure 2:**
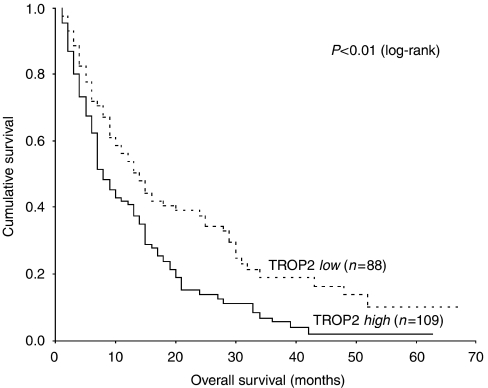
Prognostic significance of TROP2 antigen expression in 197 patients with pancreatic ductal adenocarcinomas regarding overall survival as calculated by Kaplan–Meier analysis. Low, patients with tumour (*n*=88) without TROP2 overexpression; high, patients with tumour (*n*=109) with TROP2 overexpression. Patients with low TROP2 expression had significantly better overall survival than patients with high TROP2 expression as defined by the log-rank test (*P*<0.01).

Univariate analysis showed TROP2 overexpression to be significantly correlated with histologic grading (*P*=0.01) and the presence of lymph node metastases (*P*=0.04), but not with sex, age, T stage, presence of distant metastasis, or involvement of the resection margin (Table 2). To assess the impact of clinicopathological features and TROP2 overexpression on survival, we used Kaplan–Meier analysis and the log-rank test for censored survival data. Consistent with earlier findings, early tumour stage and a negative resection margin were associated with longer overall survival (Table 1). It should be noted that TROP2 overexpression was significantly correlated with poor overall survival (*P*<0.01; Figure 2). Median overall survival time for patients with tumour presenting with and without TROP2 overexpression was 8 and 14 months, respectively. Overall survival gradually declined with increasing TROP2 scores. In the subset of patients with tumours lacking TROP2 overexpression (score 0), median overall survival time was 15 months and decreased to 14 months (score 1–4), 10 months (score 5–8) and 7 months (score 9–12) with increasing staining scores. Multivariate analysis identified TROP2 overexpression together with a negative resection margin as an independent prognostic factor for poor overall survival (Table 3a). Subsequently, a detailed analysis for the subgroup of patients who underwent surgery with curative intent (*n*=134) was performed.

### Resected cohort with curative intent

**Figure 3:**
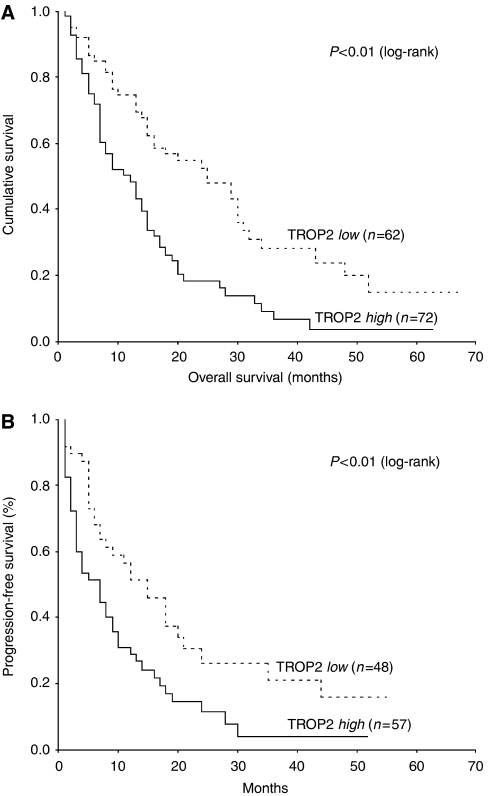
Prognostic significance of TROP2 antigen expression in the subgroup of patients who underwent surgery with curative intent regarding overall (*n*=134) and progression-free (*n*=105) survival as calculated by Kaplan–Meier analysis. Patients with tumour tissue presenting TROP2 overexpression (TROP2 high) had a significant shortened overall survival (**A**) and progression-free (**B**) interval as compared with patients with tumours lacking TROP2 overexpression (TROP2 low).

Data on relapse or disease progression were available in 78% (*n*=105) of the patients. Median overall and progression-free survival was 13 (range, 1–68) months and 6 (range, 1–56) months, respectively. Survival analyses revealed that TROP2 overexpression was significantly correlated with poor overall survival (*P*<0.01; Figure 3A) as well as a shortened progression-free interval (*P*<0.01; Figure 3B). Table 3b shows the multivariate analyses for patients treated surgically with curative intent. This model was refined by stepwise removal of variables. Regarding overall survival, TROP2 overexpression together with a negative resection margin has been identified to be an independent prognostic marker. Concerning progression-free survival, TROP2 overexpression was independent of nodal status and margin involvement by the tumour.

## Discussion

This study describes the prognostic value of TROP2 expression in pancreatic cancer. Overexpression of TROP2 was detectable in 109 (55%) of 197 tumour samples and was significantly correlated with decreased overall survival. In multivariate analyses, TROP2 overexpression was shown to be an independent prognostic biomarker for poor overall survival. Furthermore, TROP2 overexpression was significantly associated with a shortened progression-free survival in the subgroup of patients who underwent surgery with curative intent.

Both cell-surface glycoproteins, TROP2 and EpCAM, are encoded by the GA733 gene family and have been efficiently targeted by monoclonal antibodies *in vitro* as well as *in vivo* (Velders *et al*, 1998; Ruan *et al*, 2003; Wang *et al*, 2008). Similarly TROP2, EpCAM overexpression was also found to be a strong prognostic marker for poor survival in various epithelial cancer entities (Gastl *et al*, 2000; Varga *et al*, 2004; Stoecklein *et al*, 2006). Owing to its immunogenic properties in cancer patients, active and passive immunotherapeutic agents against the EpCAM antigen have been developed (Riethmuller *et al*, 1998; de Bono *et al*, 2004).

The functional role of TROP2 in carcinogenesis and tumour progression is only poorly understood. Fornaro *et al* (1995), who first cloned the human TROP2 gene, speculated that TROP2 might be a cell-surface signal transducer and might regulate tumour cell proliferation. This assumption is substantiated by the finding that the cytoplasmic domain of TROP2 contains several potential phosphorylation sites and TROP2 cross-linking antibodies can cause a transient increase in intracellular calcium levels (Basu *et al*, 1995; Ripani *et al*, 1998). Moreover, it has been shown that human cancer cells can express an oncogenic hybrid mRNA between TROP2 and cyclin D1, a master regulator of the cell cycle (Terrinoni *et al*, 2001). Interestingly, TROP2 seems to be involved in regulating the shift between cell migration and differentiation in primordium cells (Villablanca *et al*, 2006). However, its physiological ligand is still unknown. Schon *et al* (1993) were able to demonstrate that EpCAM can undergo proteolytic processing and that cleavage may activate EpCAM signalling. Similar mechanisms have been hypothesised for TROP2 (Fornaro *et al*, 1995). Recently, TROP2 expression was shown to correlate with invasive tumour phenotypes and poor prognosis (Ohmachi *et al*, 2006; Fong *et al*, 2008). In pancreatic cancer, TROP2 was previously reported to be highly expressed (Iacobuzio-Donahue *et al*, 2002), whereas Wang *et al* (2008) identified TROP2 as an oncogene and a potential therapeutic target in colon cancer. In fact, blocking experiments with anti-TROP2 antibodies revealed that TROP2 can contribute to tumour cell migration and invasion. Moreover, increasing TROP2 expression prompted an anchorage-independent growth of colon cancer cells.

The murine monoclonal antibody (mAb) RS7 reacts specifically with TROP2 and exhibits functional properties that make it an attractive tool for clinical diagnostic and therapeutic applications (Stein *et al*, 2003b). Indeed, radiolabelled RS7 has been applied for tumour imaging and revealed significant antitumour activity in several animal models (Shih *et al*, 1995; Stein *et al*, 1999c). Meanwhile, RS7 was successfully humanised and showed promising antitumour effects in an *in vivo* breast cancer model (Govindan *et al*, 2004).

In conclusion, we have demonstrated that TROP2 is a novel prognostic biomarker for pancreatic adenocarcinoma. These data must be confirmed and validated prospectively. Furthermore, TROP2 harbours the potential to become an attractive target for antibody-based therapies for the subset of patients with TROP2-overexpressing pancreatic cancer.

## Figures and Tables

**Table 1:** Patient characteristics

	**Pancreatic cancer**	
**Variable**	**(*n*) (%)**	***P*-value (log-rank test)**
*Sex (*n*=197)*		
Male	111 (56)	0.80
Female	86 (44)	
		
*Age (*n*=197)*		
Mean	65	
Median	67	
Range	37–91	
		
*T stage (*n*=176)*		
pT1	13 (7)	<0.01
pT2	52 (29)	
pT3	80 (46)	
pT4	31 (18)	
		
*Lymph node status (*n*=176)*		
Negative	65 (37)	<0.01
Positive	111 (63)	
		
*Distant metastasis (*n*=142)*		
No	117 (82)	<0.01
Yes	25 (18)	
		
*Surgical margin (*n*=128)*		
Negative	86 (67)	<0.01
Positive	42 (33)	
		
*Stage (UICC) (*n*=180)*		
IA	6 (3)	<0.01
IB	24 (13)	
IIA	34 (19)	
IIB	70 (39)	
III	20 (11)	
IV	26 (15)	
		
*Differentiation (*n*=181)*		
Well	24 (13)	0.07
Moderate	84 (47)	
Poor	73 (40)	
		
*TROP2 overexpression (*n*=197)*		
Yes	109 (55)	<0.01
No	88 (45)	

**Table 2:** Correlation of TROP2 overexpression with conventional clinicopathological parameters

		**TROP2 overexpression**		
		**No**	**Yes**	
	**Total patients (*n*)**	***n* (%)**	***n* (%)**	***P-value***
*Sex (*n*=197)*				
Male	111	51 (46)	60 (54)	0.68
Female	86	37 (43)	49 (57)	
				
*Age at diagnosis (*n*=197)*				
<65 years	84	37 (44)	47 (56)	0.88
⩾65 years	113	51 (45)	62 (55)	
				
*Differentiation (*n*=181)*				
Well	24	17 (71)	7 (29)	0.01
Moderate	84	38 (45)	46 (55)	
Poor	73	26 (36)	47 (64)	
				
*T stage (*n*=176)*				
pT1	13	5 (38)	8 (62)	0.06
pT2	52	27 (52)	25 (48)	
pT3	80	36 (45)	44 (55)	
pT4	31	7 (23)	24 (77)	
				
*Lymph node status (*n*=176)*				
No	65	34 (52)	31 (48)	0.04
Yes	111	41 (37)	70 (63)	
				
*Distant metastasis (*n*=142)*				
No	117	56 (48)	61 (52)	0.14
Yes	25	8 (32)	17 (68)	
				
*Surgical margin (*n*=128)*				
Negative	86	45 (52)	41 (48)	0.10
Positive	42	16 (38)	26 (62)	
				
*Stage (UICC) (*n*=180)*				
IA	6	4 (67)	2 (33)	0.03
IB	24	15 (63)	9 (37)	
IIA	34	15 (44)	19 (56)	
IIB	70	32 (46)	38 (54)	
III	20	3 (15)	17 (85)	
IV	26	9 (35)	17 (64)	

**Table 3:** Multivariate analyses (Cox regression) of various prognostic parameters in the total cohort and subgroup of patients with curative intent

				**Overall survival**		
				***P*-value**	**RR**	**95% CI**
*(a) Total group of patients (*n*=197)*						
T stage				0.81	1.0	0.7–1.4
Nodal status				0.94	0.9	0.5–1.6
Distant metastasis				0.71	0.7	0.1–5.2
Surgical margin				0.02	1.9	1.1–3.5
TROP2 overexpression				0.01	1.8	1.1–3.1
						

	**Progression-free survival**			**Overall survival**		
	***P*-value**	**RR**	**95% CI**	***P*-value**	**RR**	**95% CI**
*(b) Subgroup of patients treated surgically with curative intent (*n*=134)*						
T stage	0.720	0.9	0.6–1.3	0.721	0.9	0.6–1.3
Nodal status	0.545	1.1	0.6–2.1	0.764	0.9	0.5–1.5
Surgical margin	0.276	1.4	0.7–2.7	0.012	2.0	1.1–3.5
TROP2 overexpression	0.070	1.6	0.9–2.8	0.009	1.8	1.1–3.0
						
Nodal status	0.562	1.1	0.6–2.1	0.772	0.9	0.5–1.5
Surgical margin	0.301	1.3	0.7–2.6	0.012	1.9	1.1–3.3
TROP2 overexpression	0.073	1.6	0.9–2.7	0.009	1.8	1.1–3.0
						
Surgical margin	0.185	1.4	0.8–2.7	0.009	1.9	1.1–3.1
TROP2 overexpression	0.053	1.6	0.9–2.8	0.009	1.8	1.1–2.9
						
Nodal status	0.193	1.3	0.8–2.2	0.288	1.2	0.8–1.9
TROP2 overexpression	0.014	1.8	1.1–2.9	0.000	2.0	1.3–3.1

Abbreviations: CI=confidence Interval; RR=relative risk.
